# IL-6 Expression Regulates Tumorigenicity and Correlates with Prognosis in Bladder Cancer

**DOI:** 10.1371/journal.pone.0061901

**Published:** 2013-04-30

**Authors:** Miao-Fen Chen, Paul-Yang Lin, Ching-Fang Wu, Wen-Cheng Chen, Chun-Te Wu

**Affiliations:** 1 Department of Radiation Oncology, Chang Gung Memorial Hospital, Chiayi, Taiwan; 2 Chang Gung University, College of Medicine, Taoyuan, Taiwan; 3 Department of Pathology, Chang Gung Memorial Hospital, Chiayi, Taiwan; 4 Department of Urology, Chang Gung Memorial Hospital, Chiayi, Taiwan; 5 Department of Urology, Chang Gung Memorial Hospital, Keelung, Taiwan; Columbia University, United States of America

## Abstract

Identification of potential tumor markers will help stratify and identify a tumor's malignant potential and its response to specific therapies. IL-6 has been reported to be a predictor in various cancers. Therefore, the present study was performed to highlight the role of IL-6 in improving treatment and determining prognosis of bladder cancer. The human bladder cancer cell lines HT1376 and HT1197 were selected for cell and animal experiments, in which biological changes after experimental manipulation of IL-6 were explored, including tumor behavior and related signaling in bladder cancer. In addition, clinical specimens from 85 patients with muscle-invasive, and 50 with non-muscle invasive bladder cancers were selected for immunohistochemical staining to evaluate the predictive capacity of IL-6 in relation to clinical outcome. The data revealed that IL-6 was overexpressed in the bladder cancer specimens compared with non-malignant tissues at both mRNA and protein levels. Positive staining of IL-6 was significantly correlated with higher clinical stage, higher recurrence rate after curative treatment, and reduced survival rate. Tumor growth and invasive capability were attenuated when IL-6 was blocked. The underlying changes included decreased cell proliferation, less epithelial-mesenchymal transition (EMT), decreased DNA methyltransferase 1 expression and attenuated angiogenesis. In conclusion, our findings showed that IL-6 could be a significant predictor for clinical stage and prognosis of bladder cancer. Moreover, targeting IL-6 may be a promising strategy for treating bladder cancer.

## Introduction

Urinary bladder cancer represents a spectrum of neoplasms, including non-muscle invasive (NMIBC), muscle invasive, and metastatic lesions. Transitional cell carcinoma (TCC) of the bladder is the most common form of bladder cancer and is manifested in two distinct forms with different clinical and biological behaviors. Approximately 70% of patients present with non-muscle invasive tumors, while the remaining 30% present with muscle-invasive tumors. Despite good prognosis for patients with superficial disease, recurrence is common and is associated with development of muscle-invasive disease [1]. Nearly half of all patients presenting with muscle-invasive disease or those who have already progressed to this state harbor occult distant metastasis and have poor 5-year survival rate [2]. Unlike other urological cancers, bladder cancer lacks clinical useful biomarkers for predicting disease stage and clinical outcome. Therefore, molecular markers that can be used to stratify and identify a tumor's true malignant potential and its response to specific therapies are required.

Chronic inflammation often precedes or accompanies a substantial number of cancers [3]–[5]. An increase in inflammatory mediators has been shown to lead to tumor promotion, invasion, and angiogenesis [6]–[7]. Although the role of chronic inflammation in the etiology of TCC of the bladder has not been well established, there is mounting evidence that proinflammatory cytokines play critical roles in the pathogenesis of bladder cancer, such as IL-6, IL-8 and TNF- α [8]. Moreover, persistently STAT3 activation was shown to maintain constitutive NF-κB activity, thus providing evidence for the relation between oncology signaling pathways within the inflammatory microenvironment [9]. IL-6 is a major activator of STAT3 signaling pathways, and the main cytokine influencing the inflammatory response in humans [10], [11]. IL-6 signaling has been implicated in regulation of tumor growth and metastatic spread, and its level could be correlated with poor prognosis in different cancers [11], [12]. Furthermore, increased IL-6 serum levels were reported to be associated with metastasis and poor prognosis of prostate, ovarian, and bladder cancers [13]–[15]. Although there is evidence suggesting that IL-6 may be a critical factor in various malignancies, its role in bladder cancer remains unclear. Therefore, in the present study, we focused on the underlying mechanisms of IL-6 and its possible usefulness for addressing the need of aggressive treatment for bladder cancer.

## Materials and Methods

### Patient characteristics

The Institutional Review Board of Chang Gung Memorial Hospital approved the present study (Permit Number: 99-0207B). The written consents were signed by the patients for their specimen and information to be stored in the hospital and used for research. A total of 85 patients with muscle-invasive bladder TCC (39 with stage T2 and 46 with stages T3 –T4) who completed a course of definite chemoradiotherapy (CCRT) treatment were enrolled in the study. On completion of CCRT, patients underwent a repeat computed tomography scans (CT) and cystoscopy examination to determine the response to treatment. Patients were observed at 3-month intervals for the first 2 years and every 6 months thereafter. The follow-up for patients was continued until their death, and the end points were overall survival (OS), progression-free survival, failure pattern and response to definite CCRT. Disease progression was defined as documented local recurrence or distant metastases. Analyses were performed using SPSS version 17.0.

### Immunohistochemical (IHC) staining

In addition to tissue specimens collected from the 85 patients with muscle-invasive bladder cancer, bladder tissue specimens were collected for immunohistochemical (IHC) staining from 17 patients suffering from bladder carcinoma with distant metastasis, 50 NMIBC patients, and 40 non-malignancy patients. Formalin-fixed, paraffin-embedded tissues obtained by transurethral resection in the diagnosis were cut into 5 µm sections, and mounted on slides for IHC staining. For histological evaluation of IL-6 staining, the staining was scored independently by two observers who were blind to the clinical outcome of patients; discordant scores were reviewed, and a consensus was reached. In the present study, a criterion of more than 10% positive staining of tumor cells was considered positive on IHC scoring, which was defined by receiver operating characteristic (ROC) curve analysis.

### Cell culture and reagents

HT137 and HT1197, human bladder cancer cell lines, were obtained from the American Type Culture Collection (ATCC). The IL-6-neutralizing antibody was obtained from R&D Systems (Minneapolis, MN) and the IL-6-GFP silencing vector (human IL6 shRNA constructs in retroviral GFP vector) and GFP-control vector (Non-effective scrambled shRNA cassette in retroviral GFP vector) were obtained from Origene Technologies, Inc. (Rockville, MD).

### Tumor xenograft model (ectopic and orthotopic)

This study was carried out in strict accordance with the recommendations in the Guide for the Care and Use of Laboratory Animals as promulgated by the Institutes of Laboratory Animal Resources, National Research Council, U.S.A. The protocol was approved by the Committee on the Ethics of Animal Experiments of Chang Gung Memorial Hospital (Permit Number: 2010070201). Eight-week-old female athymic nude mice were used as the xenograft tumor implantation model. In the ectopic tumor implantation model, cells (5×10^6^ tumor cells were injected subcutaneously per implantation, five animals per group) were implanted into the bilateral dorsal gluteal region. Tumor size was measured every three days after implantation (day 0). The tumor volume was calculated assuming an ellipsoid shape. In the orthotopic tumor implantation model, we performed intravesicular instillation of canccer cells as described previously (five animals per group). The extent of orthotopic tumor invasion was measured after implantation at the indicated times. The effect of IL-6 stimulation was also investigated *in vivo*. For the treated group, an intraperitoneal injection of IL-6 (60 or 100 ng per mouse, 3 times per week) was started one day before tumor implantation.

#### Cell migration and cell invasion assay

Capacity for cell invasion was determined by Cell Invasion Assay (Trevigen, Gaithersburg, MD). The top chambers were pre-coated with basement membrane extract (derived from EHS tumor and provided in the kit). After incubation for 24 h, the number of cells in the bottom chamber was determined by measuring the fluorescent anion calcein released from intracellular calcein acetoxymethylester. To validate experiments on cell migration, scratch assays were also done. A 2 mm wide scratch was drawn across each cell layer using a pipette tip. The plates were photographed at the times indicated.

### Immunofluorescence (IF) staining

Cells were seeded onto glass coverslips at 5×10^4^ cells/ml in 6- well plates for immunofluorescence (IF) staining with or without treatment. At the specified times after treatment, cells were fixed with 2% paraformaldehyde for 5 minutes, and washed in PBS with Tween-20 (PBST). Slides were incubated for 1 h at room temperature with antibodies against E-cadherin and IL-6, followed by incubation with Texas Red-conjugated secondary antibody and counterstaining with 4′,6-diamidino-2-phenylindole (DAPI).

### Real-time reverse transcription-polymerase chain reaction (RT-PCR)

Real-time RT-PCR was performed on RNA extracted from cells and tissue specimens (six cancer tissue specimens and six non-malignant tissues; two specimens in each lane). The primer sequences were as follows: (forward and reverse, respectively) 5′-GTTCTTCCTCCTGGAGAATGTCA-3′ and 5′-GGGCCACGCCGTACTG-3′ for DNMT-1; 5′-TACATCCTCGACGGCATCTC-3′ and 5′-GCTACATTTGCCGAAGA- -GCC-3′ for IL-6. A β-actin primer set was used as a loading control. The optimized PCR was performed on an iCycler iQ multicolor real-time PCR detection system. Significant fluorescent PCR signals from carcinoma tissue were normalized relative to the mean value of signals obtained from non-malignant tissues.

### Enzyme-linked immunosorbent assay (ELISA) for IL-6 levels *in vitro* and *in vivo*

Urine specimens were obtained from 60 patients with bladder cancers (25 from patients with NMIBC, 35 from those with muscle-invasive disease), and 20 samples from patients without evidence of malignancy. Sample of 10 ml of fresh urine was collected from each subject, and subsequently centrifuged at 3000 rpm for 5 minutes. At the time of analysis urine aliquots were defrosted and urinary IL-6 were measured in the supernatants. Commercially available ELISA assay (HS human IL-6 immunoassay kit; R&D Systems) was used to measure levels of urinary IL-6. The assays were conducted according to the manufacturer's instructions. Calibration curves were prepared using purified standards for each protein assessed. To test IL-6 level in cellular supernatant, the cells were cultured with 1 ml serum-free medium for 24 h in 6-well plate. The medium was collected and clarified by centrifugation at 3000 g. IL-6 level in the supernatant was detected by ELISA assay.

### Statistical analysis

Survival probabilities were analyzed using the Kaplan–Meier method. Survival was calculated from the date of treatment started to the date of death or the most recent follow-up. The significance of differences between groups was assessed using the log-rank test. All statistical tests were two-sided, with *p*<0.05 taken to indicate significance. Significance of difference between samples was determined using Student's t-test. Data are presented as mean±standard error of the mean (SEM). Three repeats were carried out for evaluating each experiment, and repeat the entire set of experiments at least twice. A probability level of p<0.05 was adopted throughout to determine statistical significance unless otherwise stated.

## Results

### IL-6 expressions in patients with bladder cancers

**Figure 1:**
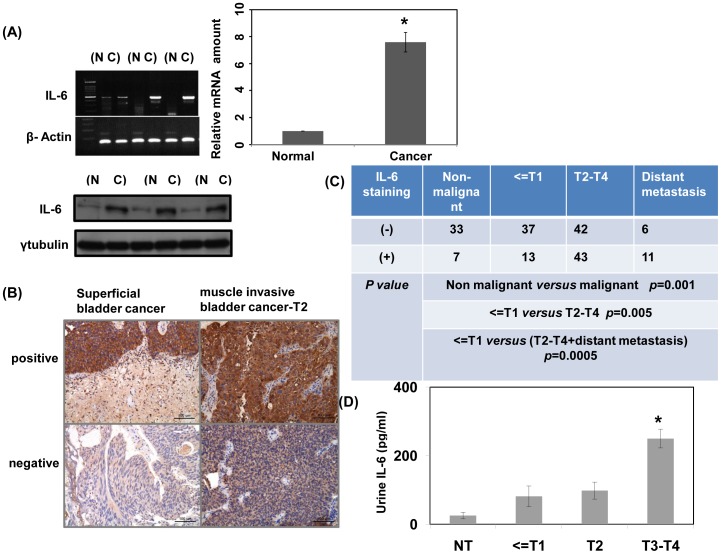
Levels of IL-6 in bladder cancer. A. Levels of IL-6 were examined in six specimens (paired cancer (C) and adjacent non-malignant tissue (N); two specimens in a lane) by RT-PCR and Western blotting analysis. For real-time RT-PCR analysis, the y-axis shows the ratio of IL-6 in cancer tissue divided by that in the non-malignant specimen. Columns, mean of three separate experiments; Bars, standard deviation (SD); *, *P<*0.05. B. Immunohistochemical staining of human bladder cancer specimens with anti-IL-6 antibody. Representative slides are shown. C. The IHC data showed that IL-6 levels were significantly correlated with clinical stage. D. Urinary IL-6 levels of patients were examined by ELISA analysis. Columns, mean of three separate experiments; Bars, SD; *, *P<*0.05. (NT, patients with non-malignant diseases).

The level of IL-6 in tissue specimens (six paired cancer and adjacent nonmalignant tissue specimens) was examined using mRNA and protein analyses. As shown in Figure 1A, bladder cancer specimens expressed substantially higher level of IL-6 than non-malignant tissues. IHC analysis for bladder TCC (Fig. 1B and Table 1) indicated positive staining for IL-6 in 51% of T2 –T4 bladder cancer tissues [28% (11/39) in T2 vs. 70% (32/46) in T3 –T4; *P* = 0.0001]. In addition, 65% (11/17) of the more advanced tumors (patients with distant metastases) showed positive staining for IL-6, but only 17.5% (7/40) of non-malignant bladder tissues and 26% (13/50) of early-stage tumors (CIS or T1) expressed IL-6. As shown in Figure 1C, positive staining for IL-6 was significantly correlated with clinical stage (T2 –T4 vs. T1 and CIS; *P* = 0.005). Moreover, the urinary levels of IL-6 were examined by ELISA analysis. The mean IL-6 levels in urine samples from patients with NMIBC (81±30.8 pg/mL) and T2 muscle-invasive bladder cancer (98±24 pg/mL) were slightly higher than those without malignant disease (25.14±9.71 pg/mL), but the differences were not statistically significant (Fig. 1D). Urinary IL-6 levels were significantly elevated in patients with T3 –T4 local-advanced bladder cancers (250±27 pg/mL) compared to those in patients with ≤T2 bladder cancer or non-malignant disease (*P* = 0.01).

**Table 1:** Baseline characteristics of patients.

	No. of	patients	
	IHC-IL-6 (−)	IHC-IL-6 (+)	*p* value
**Patients with**			
**non-muscle invasive**	37	13	
**bladder cancer**			
**Age**			0.52
Median	69.9	68.2	
Range	41.9–89.5	51.9–83.4	
**Gender**			0.94
Male	26	9	
Female	11	4	
**IHC–DNMT1**			0.0002^*^
(+)	4	8	
(−)	33	4	
**Clinical stage**			0.19
Ta	13	2	
Tis&T1	24	11	
**Patients with muscle-**			
**invasive bladder cancer**	42	43	
**Age**			0.554
Median	70.0	73.9	
Range	46.5–88.2	48.5–92.5	
**Gender**			0.19
Male	28	34	
Female	14	9	
**IHC-DNMT1**			0.012^*^
(+)	17	29	
(−)	25	14	
**Clinical stage**			0.0001^*^
T2	28	11	
T3–T4	14	32	
**Histologic grade**			0.097
Low-intermediate	19	12	
High	23	31	
**LN involvement**			0.039^*^
Negative	37	30	
Positive	5	13	
**RT dose (cGy)**			0.401
mean	5832	5736	
median	5940	5940	
**Response to**			
**definite CCRT**			0.056
CR (+)	35	28	
CR (−)	7	15	
**Disease status**			0.0001^*^
Control	32	13	
Failure (LR+DM)	10	30	

Abbreviations: CCRT  = concurrent chemotherapy and radiotherapy; LN = lymph node; CR = complete response; LR = local recurrence; DM = distant metastasis.*, *P<*0.05.

### Role of IL-6 in tumor growth and invasion

**Figure 2:**
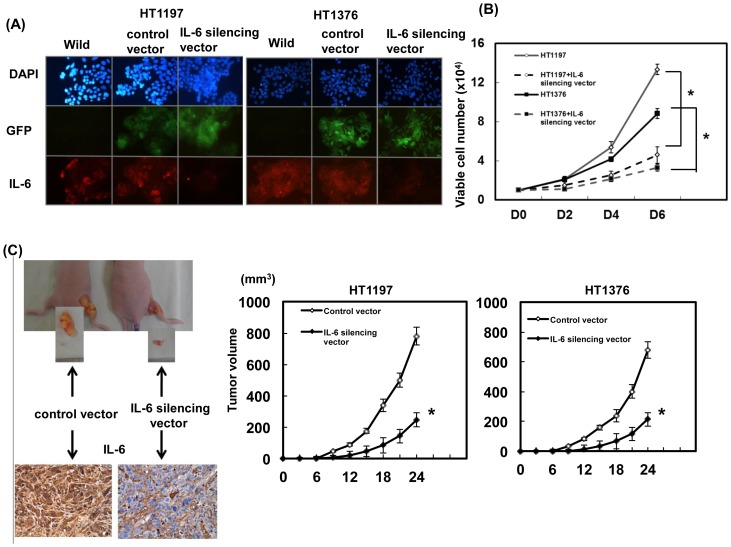
Role of IL-6 in tumor cell growth. A. Effects of the IL-6-GFP silencing vector on IL-6 level in HT1197 and HT1376 cells as demonstrated by immunofluorescence analysis. Representative micrographs are shown, with the respective immunofluorescent colors (DAPI, blue; GFP, green; IL-6, red). IL-6 levels were significantly decreased by the IL-6-GFP silencing vector compared with the control-GFP vector. B. Effects of the IL-6 silencing vector on he proliferation rates of HT1197 and HT1376 cancer cells. The same number of cells (10^4^) was plated in each plate on day 0 and allowed to grow in their respective cultures. We counted the numbers of viable cells after incubation for 2, 4, and 6 days. The y-axis represents the viable cell number. Point, mean of three separate experiments. Bars, SD. *, *P<*0.05. C. Effects of IL-6 inhibition on xenograft tumor growth. Each point represents the mean of three separate experiments; bars, SD; *, *P<*0.05. Expression of IL-6 was also evaluated by immunochemical staining of xenografts. Representative slides are shown at×400 magnification.

**Figure 3:**
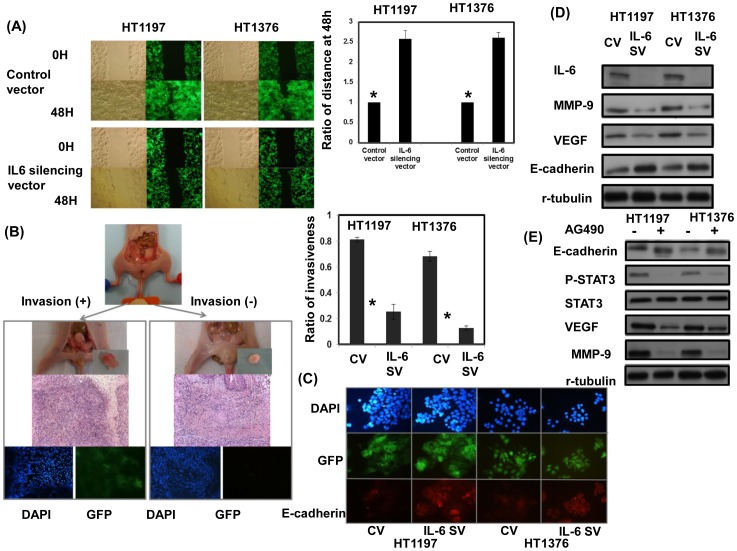
Effects of IL-6 inhibition on aggressive tumor behavior and EMT changes. A. The invasive capacity of bladder cancer cells with or without the IL-6 silencing vector was evaluated by migration scratch assays. The results from representative slides are shown. Column, mean of three separate experiments. Bars, SD. *, *P<*0.05. B. The invasive capacity of bladder cancer cells with or without the IL-6 silencing vector was evaluated by murine orthotopic tumor implantation. The representative slides and quantitative data are shown. The y-axis represents the ratio of mice presenting intravesicular tumors, normalized to that received orthotopic tumor implantation. The IL-6 silencing vector decreased the rate of tumor implantation in the bladder and was associated with a smaller tumor size. Column, mean of three separate experiments. Bars, SD. *, *P<*0.05. (CV, cells transfected with the control vector; IL-6 SV, cells transfected with the IL-6 silencing vector). C. Change in E-cadherin in cells was evaluated and the representative micrographs are shown, with the respective immunofluorescent colors (DAPI, blue; GFP, green; E-cadherin, red). D. Change in EMT-associated proteins in transfectants was evaluated by Western blotting analysis (CV, cells transfected with the control vector; IL-6 SV, cells transfected with the IL-6 silencing vector). E. Change in EMT-associated proteins in cells treated with JAK inhibitor- AG490.

To investigate whether IL-6 plays a role in the aggressive behavior of bladder cancer, HT1197 and HT1376 cells were transfected with the IL-6-GFP silencing vector. As shown in Figure 2A, the IL-6 silencing vector significantly decreased IL-6 expression in both cell lines. As determined by viable cell counts over six days (Fig.2B), the IL-6 silencing vector significantly attenuated the proliferation rate of HT1197 and HT1376 cells. Furthermore, using xenograft tumors model, inhibition of IL-6 resulted in slower tumor growth *in vivo* (Fig. 2C). The data demonstrate that the IL-6 silencing vector significantly inhibited the growth rate of bladder cancer cells. Additionally, IL-6 silencing vector significantly attenuated the invasive capacity of bladder cancer cells as demonstrated using migration scratch assays [16] and invasion assay *in vitro* (Fig. 3A, Fig. S1). An orthotopic tumor implantation technique was used to examine the effects of the IL-6 silencing vector on invasive capability *in vivo* (Fig. 3B). Sixteen mice received intravesicular instillation of each bladder cancer cell line. After 28 days, 13 mice (81%) instilled with HT1197 cells, four (25%) instilled with HT1197 cells plus IL-6 silencing vector, 11 (69%) with HT1376 cells, and two (13%) with HT1376 cells plus IL-6 silencing vector developed intravesicular tumors. The data indicated that the IL-6 silencing vector decreased invasive capability *in vivo*.

### Role of IL-6 in EMT changes

EMT is a key event in invasiveness [17], and we examined whether this is the mechanism underlying the aggressive behavior of IL-6-positive bladder cancer. As shown in Figure 3C-D, the IL-6 silencing vector increased expression of E-cadherin associated with decrease in vascular endothelial growth factor (VEGF) and matrix metallopeptidase 9 (MMP-9) expressions in tumor cells [18]. It has been reported that IL-6 is a major activator of STAT3 signaling, and the activation of STAT3 signaling plays a role in the induction of aggressive tumor behavior and EMT changes in cancer [19]. When we blocked STAT3 activation with JAK inhibitor-AG490, the EMT-related proteins were decreased (Fig. 3E). These observations suggested that the increased aggressive tumor behavior and EMT changes induced by IL-6 might be mediated by STAT3 activation, a part at least.

### Effects of IL-6 on angiogenesis

**Figure 4:**
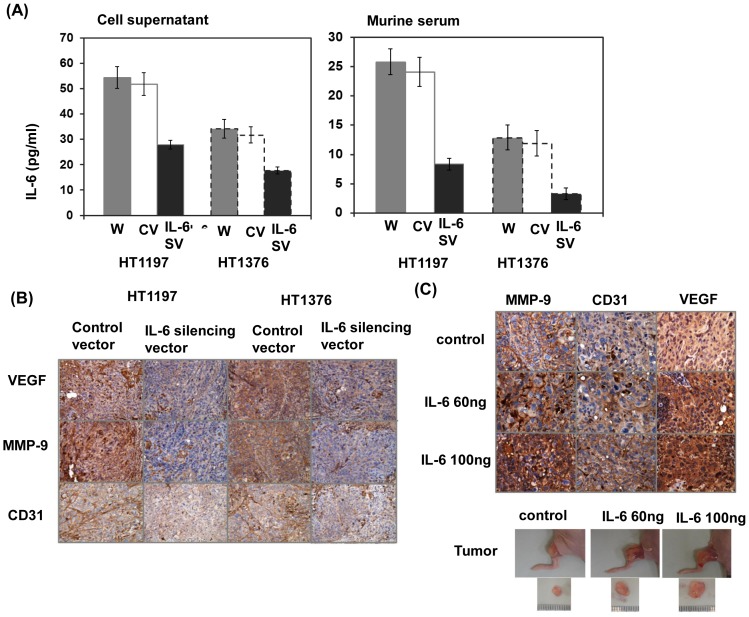
IL-6 is linked with the induction of angiogenesis. A. The level of IL-6 in cell culture supernatants and serum of mice bearing tumors with or without the IL-6 silencing vector was examined by ELISA *in vitro* and *in vivo*. Column, mean of three separate experiments. Bars, SD. *, *P<*0.05. B. Change in vascular endothelial growth factor (VEGF) and matrix metallopeptidase 9 (MMP-9), and CD31 in tumor xenografts was evaluated by immunohistochemical staining. The results from representative slides are shown. C. Effect of IL-6 on xenograft tumor growth and the induction of angiogenesis was evaluated in mice bearing tumors for 2 weeks. Tumor cells were injected subcutaneously into the mice with or without IL-6 injection as indicated in Materials and Methods, and tumor growth examined 2 weeks later. Immunohistochemistry using MMP-9, CD31, and VEGF stained- representative slides were demonstrated.

ELISA data revealed that IL-6 silencing vector clearly attenuated IL-6 secretion in cell culture supernatants and serum from mice after 28 days of tumor implantation (Fig. 4A). CD31-mediated endothelial cell-cell interactions are involved in angiogenesis [20]. Figure 4B showed that IL-6 silencing vector attenuated angiogenesis demonstrated by the staining of CD31 and VEGF. To further examined whether circulating IL-6 facilitating the induction of angiogenesis, an intraperitoneal injection of IL-6 (60 or 100 ng per mouse, 3 times per week) was started one day before tumor implantation. As shown in Figure 4C, IL-6 triggered angiogenesis and endothelial tube formation within the tumor by the staining of VEGF, MMP-9 and CD31 in mice bearing tumors for 2 weeks. Therefore, induction of angiogenesis may be one of the mechanisms responsible for tumor promotion by IL-6.

### Relationship between expression of IL-6 and DNMT1 in bladder cancer

**Figure 5:**
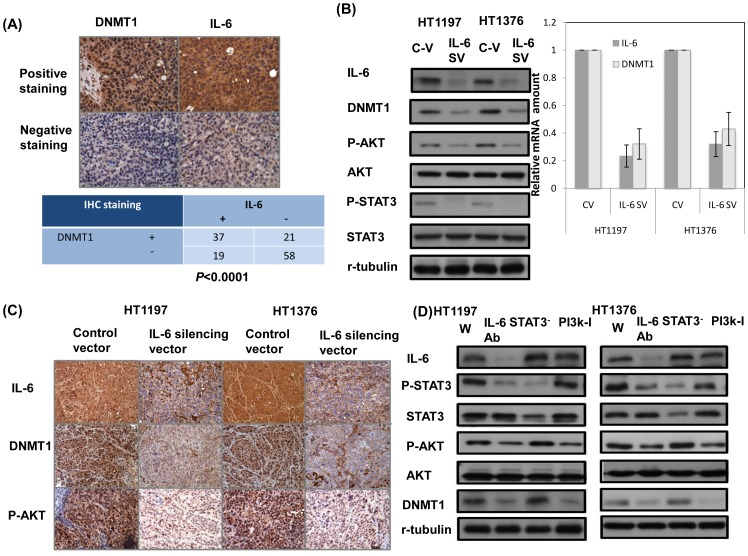
DNMT1 is linked to activated IL-6 signaling via Akt phosphorylation. A. IL-6 level was positively correlated with DNMT1 expression in human bladder cancer specimens (*P<*0.0001). Representative slides of a selected tumor specimen positively staining for both IL-6 and DNMT1, and another tumor specimen negative for both IL-6 and DNMT1 are shown at ×400 magnification. B. Effect of IL-6 on the level of DNMT1, p-AKT, and p-STAT3 was examined by Western blotting (CV, cells transfected with the control vector; IL-6 SV, cells transfected with the IL-6 silencing vector). C. Effect of IL-6 on the level of DNMT1 and p-AKT was evaluated by immunohistochemical staining, and the results from representative slides are shown. D. Effect of IL-6 inhibition by IL-6-neutralizing antibody, p-AKT inhibition by PI3K inhibitor, and p-STAT3 inhibition by STAT3 siRNA on the level of DNMT1 was examined by Western blotting (W, wild-type; IL-6^−^, cells treated with IL-6-neutralizing Ab; STAT3^−^, cells treated with Stat3 siRNA; PI3K-I, cells treated with PI3K inhibitor.

As we reported previously [21], higher DNMT1 levels were associated with aggressive tumor behavior and EMT changes in bladder cancers. We found there was a significant correlation between positive staining for IL-6 and DNMT1 on IHC staining of bladder cancer specimens (Fig. 5A). By mRNA and protein analysis, decreased IL-6 resulted in inhibited DNMT1 associated with attenuated STAT3 and Akt activation (Fig. 5B-C). We further examined whether the IL-6 inhibition decreased DNMT1 expression via inhibition of AKT using a PI3K inhibitor-LY294002 [22], or down regulation of STAT3 with STAT3 short interfering RNA (siRNA). As shown in Figure 5D, inhibition of Akt phosphorylation, but not decreased p-STAT3, significantly attenuated DNMT1 expression. Therefore, it is suggested that activation of AKT might be responsible to the increased DNMT1 in IL-6-positive bladder cancers.

### IL-6 is related to clinical outcome of bladder TCC

**Figure 6:**
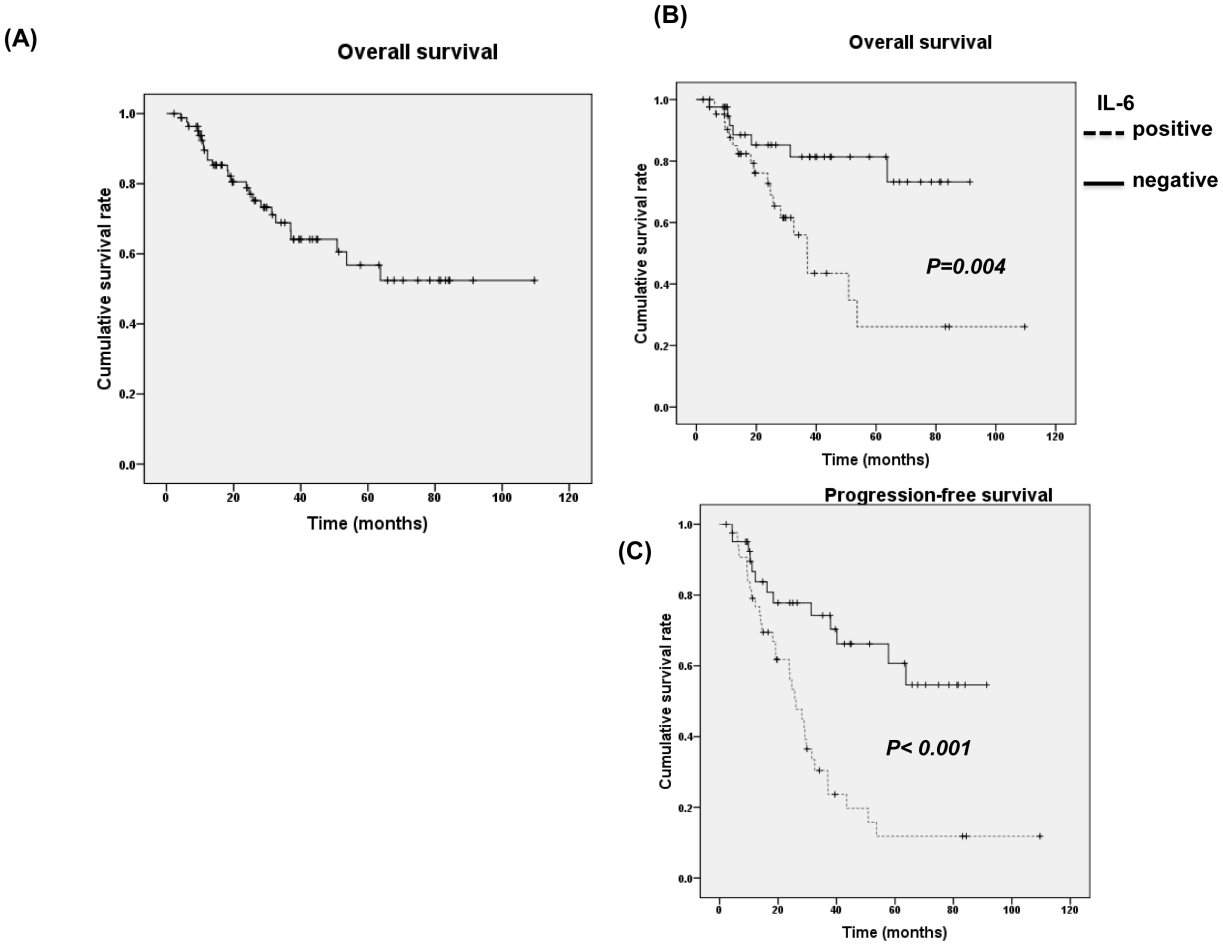
Effects of IL-6 on survival. A survival difference was demonstrated in accordance with positive staining of IL-6. The Kaplan–Meier overall survival curves showed that patients with higher levels of IL-6 expression had shorter survival periods.

The median progression-free survival time in the 85 patients completed definite CCRT treatment was 36.97 months. By univariate analysis, positive staining for IL-6 was significantly related to higher clinical stage, higher disease failure rate after definite treatment and shorter survival time (Table 1 and Figure 6). The findings strongly underscore the contribution of IL-6 to the prognosis in bladder cancer.

## Discussion

We demonstrated that IL-6 was expressed at higher levels in bladder TCC than in non-malignant tissues. Moreover, positive staining for IL-6 was preferentially associated with muscle-invasive bladder TCC relative to lower stage Ta –T1 disease. Urinary levels of IL-6 were also significantly elevated in patients with locally advanced bladder TCC compared to patients with NMIBC. Therefore, IL-6 expression might be related to a more malignant phenotype.

To investigate whether IL-6 was responsible for the aggressive behavior of bladder TCC, IL-6 was suppressed in bladder cancer cells by stable transfection with a silencing vector. Data revealed that inhibiting IL-6 resulted in decreased bladder tumor growth *in vitro* and *in vivo*. Moreover, the IL-6 silencing vector significantly attenuated the invasive ability detected in cellular invasion assays and mouse orthotopic models. IL-6 is a major activator of JAK/STAT3 signaling [10], [11], and activated STAT3 signaling has been reported to contribute to oncogenesis by promoting proliferation and EMT [18], [23]. Moreover, STAT3 activation has been shown a role in predisposing urothelial basal cells toward the CIS progression pathway into invasive bladder cancer [24]. Our data revealed that inhibition of IL-6 attenuated STAT3 activation associated with increased E-cadherin and decreased VEGF and MMP-9 expressions. Increased expressions of VEGF and MMP-9 are reported to correlate with EMT change and poor prognosis of bladder cancer [25], [26]. Therefore, it is likely that STAT3 activation plays a role in IL-6 transmitting to downstream targets that regulate EMT and invasiveness.

We showed that IL-6 levels in serum and urine were elevated in a subgroup of patients with muscle-invasive bladder cancer, consistent with other research [13], [27]. Angiogenesis is one of the mechanisms that promote tumor progression, and the expression of angiogenic factors is suggested to have predictive value for treatment response and outcome in patients with cancer [28]. Furthermore, IL-6 has been reported to play multiple functions in angiogenesis and vascular modeling [29], and increase angiogenesis by transcriptional of VEGF and MMP-9 in STAT3-dependent manner. STAT3 activation was demonstrated to modulate the expression of genes that mediate angiogenesis; *e*.*g*., VEGF [30]. Accordingly, the links between IL-6, angiogenesis, and promotion of bladder cancer in tumor-bearing mice were further investigated in the present study. Using a xenograft tumor model, our data demonstrated that IL-6 level positively linked with angiogenesis and STAT3 activation. These findings suggested that the induction of angiogenesis mediated by STAT3 activation may be one of the mechanisms underlying the aggressiveness of IL-6-positive bladder cancer.

We previously reported [21] that DNMT1 could be a significant clinical predictor of bladder cancer. Studies have identified that DNMT1 expression may be directly altered by pro-inflammatory cytokines such as IL-6 in some types of malignancies [5], [31], [32]. A positive correlation between IL-6-positive samples and nuclear staining for DNMT1 was found by IHC analysis in the present study. The relationship between IL-6/STAT3 signaling and DNMT1 in bladder cancer was further examined to see whether regulation of IL-6/STAT3 signaling results in changes of DNMT1 expression. The mRNA and protein analysis revealed that inhibited IL-6 signaling suppressed nuclear DNMT1 expression associated with decreased p-AKT and p-STAT3. However, directly inhibiting STAT3 by STAT3 siRNA had no obvious effect on DNMT1 expression. Phosphorylation of Akt kinase has been reported to be the mechanism responsible for enhanced expression of DNMT1 stimulated by IL-6 [33], [34]. When we blocked phosphoinositide 3 kinase/Akt signaling using the specific inhibitor LY294002, the attenuation of AKT activation was associated with decreased DNMT1. We therefore suggest that activated IL-6 signaling enhanced activation of DNMT1 is mediated by activation of Akt in bladder TCC.

**Figure 7:**
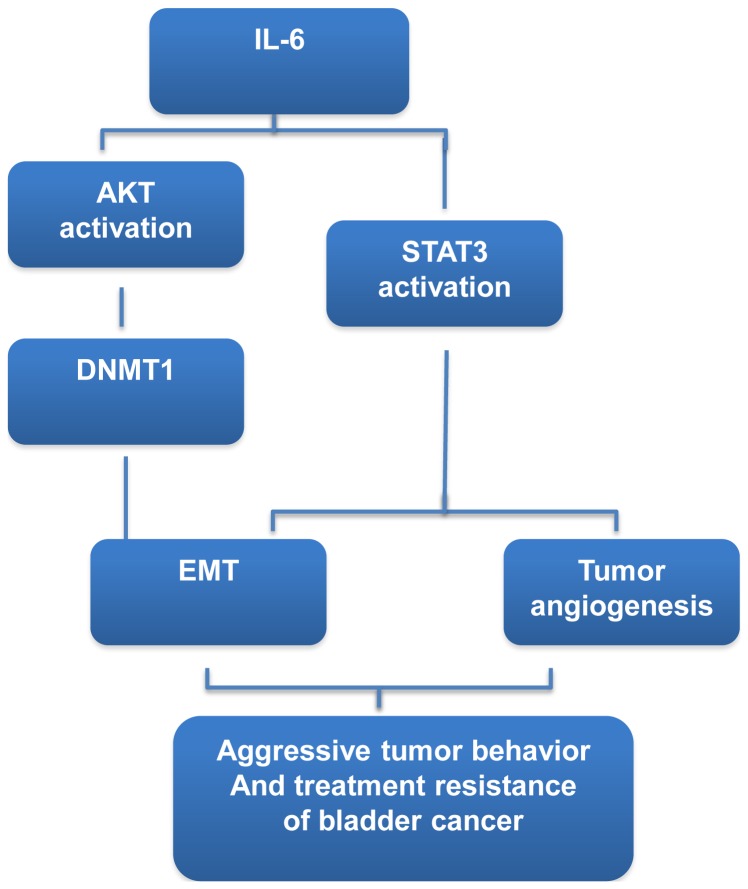
IL-6 signaling pathway in bladder cancer.

Identification of potential factors has important implications for the development and selection of molecular targets in cancer therapy. Our experimental data indicated that the level of IL-6 is important for the aggressive tumor behavior seen in bladder cancer. Therefore, we further examined the predictive power of IL-6 regarding the clinical outcome of bladder TCC after definite CCRT. Our data showed that enhanced expression of IL-6 was significantly associated with a lower complete response rate after treatment, a higher disease failure rate and a shorter survival period, demonstrating a role of IL-6 in predicting prognosis. The data obtained from the present study revealed that increased IL-6 production is critical in tumor aggressiveness and prognosis of bladder cancer. We outlined the main signaling pathways that are thought to link IL-6 to bladder cancer (Fig. 7).

In addition to IL-6, several cytokines were reported to be important in studies of bladder cancer. TNF-α was shown to stimulate bladder cancer cells to produce MMP-9, which has been implicated in tumor invaαsion and metastasis [35]. Mian et. al. reported that IL-8 blockade significantly inhibited the expressions of MMP-2 and MMP-9, resulting in decreasing invasion [36]. Moreover, increased expression of IL-8 is correlated with poor prognosis of bladder cancer [37]. Inflammation can be considered as enabling for its contribution to the acquisition of core hallmark capabilities. The biological mechanisms linking tumor aggressiveness in IL-6, IL-8 and TNF- α are not clearly understood. The issue needs further investigation in future.

Our study has some limitations. First, we retrospectively examined the predictive value of IL-6 in bladder cancer patients only by the fraction of positive staining. Also, it is a retrospective analysis of a population with different clinical stages from a single institution. Therefore, further investigations of the levels of IL-6 in patients with different stages in a prospective trial are needed.

Taken together, our findings suggest that IL-6 is crucial for aggressive tumor growth, and the clinical outcome of bladder cancer after definite radiotherapy. The data support the emerging hypothesis that IL-6 is a clinically significant prognostic predictor and may represent a suitable target for bladder cancer treatment.

## Supporting Information

Figure S1 **IL-6 inhibition attenuated the invasion capacity.** The invasive capacity in bladder cancer cells with or without IL-6 silencing vector was evaluated. The results are shown by representative slides and quantitative data. Quantification of invasion ability was counting the number of invading cells for each condition. The y-axis represents the ratio normalized by the value of the respective cell line under control condition. Column, mean of three separate experiments; Bar, SD. *, *P*<0.05
(TIF)
