# Correction: IL-6 Expression Regulates Tumorigenicity and Correlates with Prognosis in Bladder Cancer

**DOI:** 10.1371/journal.pone.0155774

**Published:** 2016-05-16

**Authors:** Miao-Fen Chen, Paul-Yang Lin, Ching-Fang Wu, Wen-Cheng Chen, Chun-Te Wu

There are errors in Fig 2A of the published article. Several panels representing the HT1197 control vector in Fig 2A were accidentally duplicated from panels in Fig 3C. A correct Fig 2A is available in S1 Fig of this correction. The underlying images for the HT1197 panels are also included as Supporting Information.

**Fig 2. Role of IL-6 in tumor cell growth.** A. Effects of the IL-6-GFP silencing vector on IL-6 level in HT1197 and HT1376 cells as demonstrated by immunofluorescence analysis. Representative micrographs are shown, with the respective immunofluorescent colors (DAPI, blue; GFP, green; IL-6, red). IL-6 levels were significantly decreased by the IL-6-GFP silencing vector compared with the control-GFP vector.

## Supporting Information

S1 Fig Correct Fig 2A. (TIF)

S2 Fig Wild DAPI. CT-400x-D1.
(TIF)

S3 Fig Control Vector DAPI. IL-6-CV-400x-D.
(TIF)

S4 Fig IL-6 Silencing Vector DAPI. IL-6-SV-400x-D.
(TIF)

S5 Fig Wild GFP. CY-400x-F1.
(TIF)

S6 Fig Control Vector GFP. IL-6-VC-400x-F.
(TIF)

S7 Fig IL-6 Silencing Vector GFP. IL-6-SV-400x-F.
(TIF)

S8 Fig Wild IL-g. CT-400x-T1.
(TIF)

S9 Fig Control Vector IL-6. IL-6-CV-400x-T.
(TIF)

S10 Fig IL-6 Silencing Vector IL-6. IL-6-SV-400x-T.
(TIF)
