# Supplementary figures and images for: IL-6 Expression Regulates Tumorigenicity and Correlates with Prognosis in Bladder Cancer
Source: PLoS One. 2013 Apr 30;8(4):e61901. doi: 10.1371/journal.pone.0061901 (PMC3640078; doi:10.1371/journal.pone.0061901)

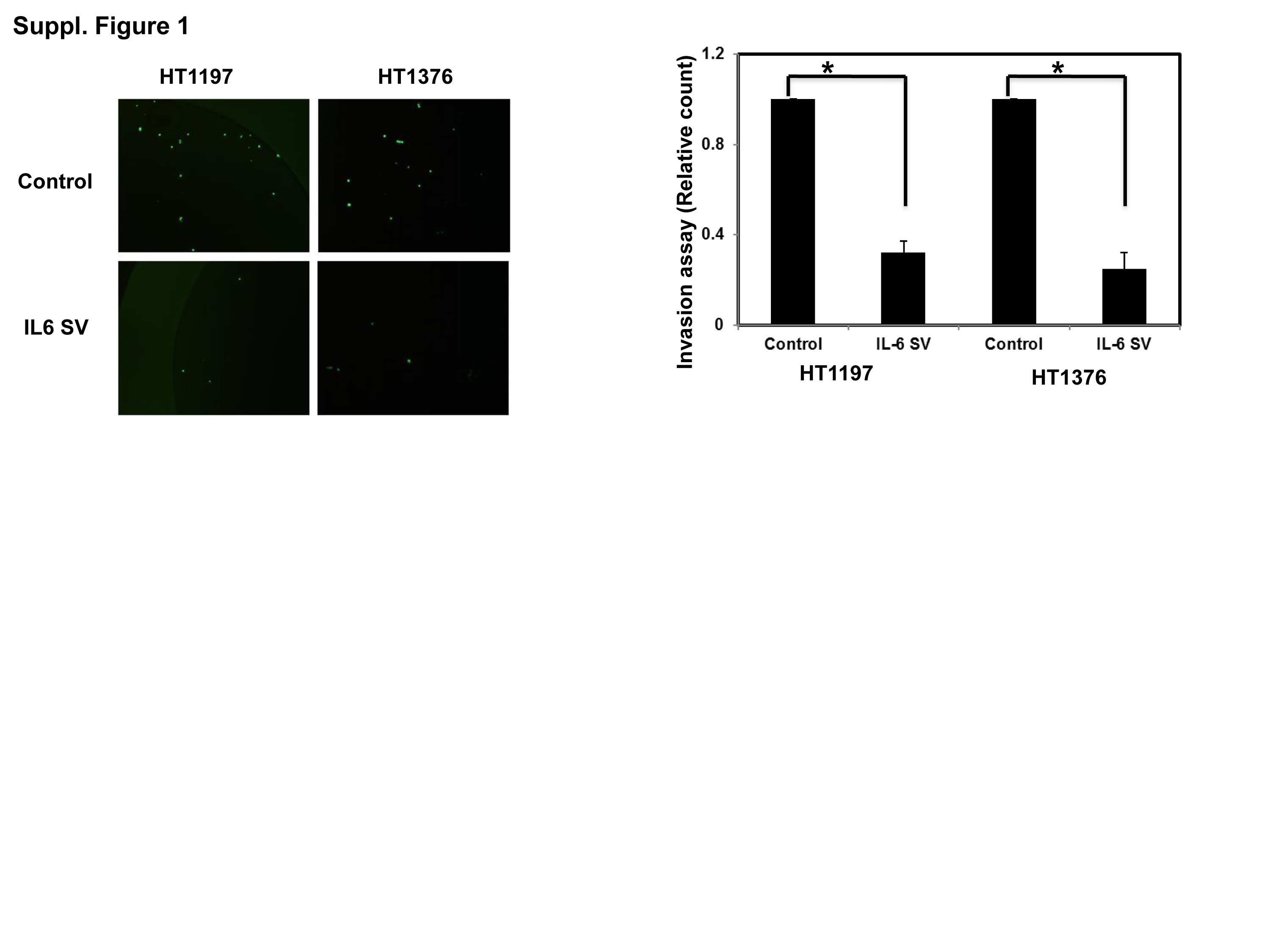

Supplement: Figure S1 — IL-6 inhibition attenuated the invasion capacity. The invasive capacity in bladder cancer cells with or without IL-6 silencing vector was evaluated. The results are shown by representative slides and quantitative data. Quantification of invasion ability was counting the number of invading cells for each condition. The y-axis represents the ratio normalized by the value of the respective cell line under control condition. Column, mean of three separate experiments; Bar, SD. *, P<0.05 (TIF) [file pone.0061901.s001.tif]
