# Supplementary figures and images for: Correction: IL-6 Expression Regulates Tumorigenicity and Correlates with Prognosis in Bladder Cancer
Source: PLoS One. 2016 May 16;11(5):e0155774. doi: 10.1371/journal.pone.0155774 (PMC4868296; doi:10.1371/journal.pone.0155774)

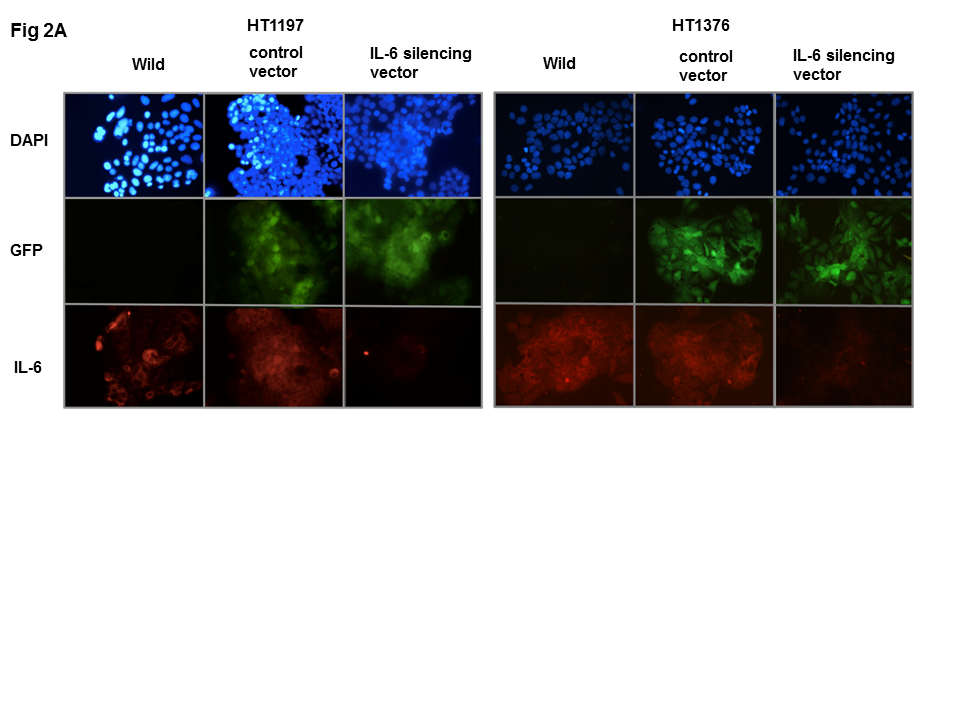

Supplement: S1 Fig — (TIF) [file pone.0155774.s001.tif]

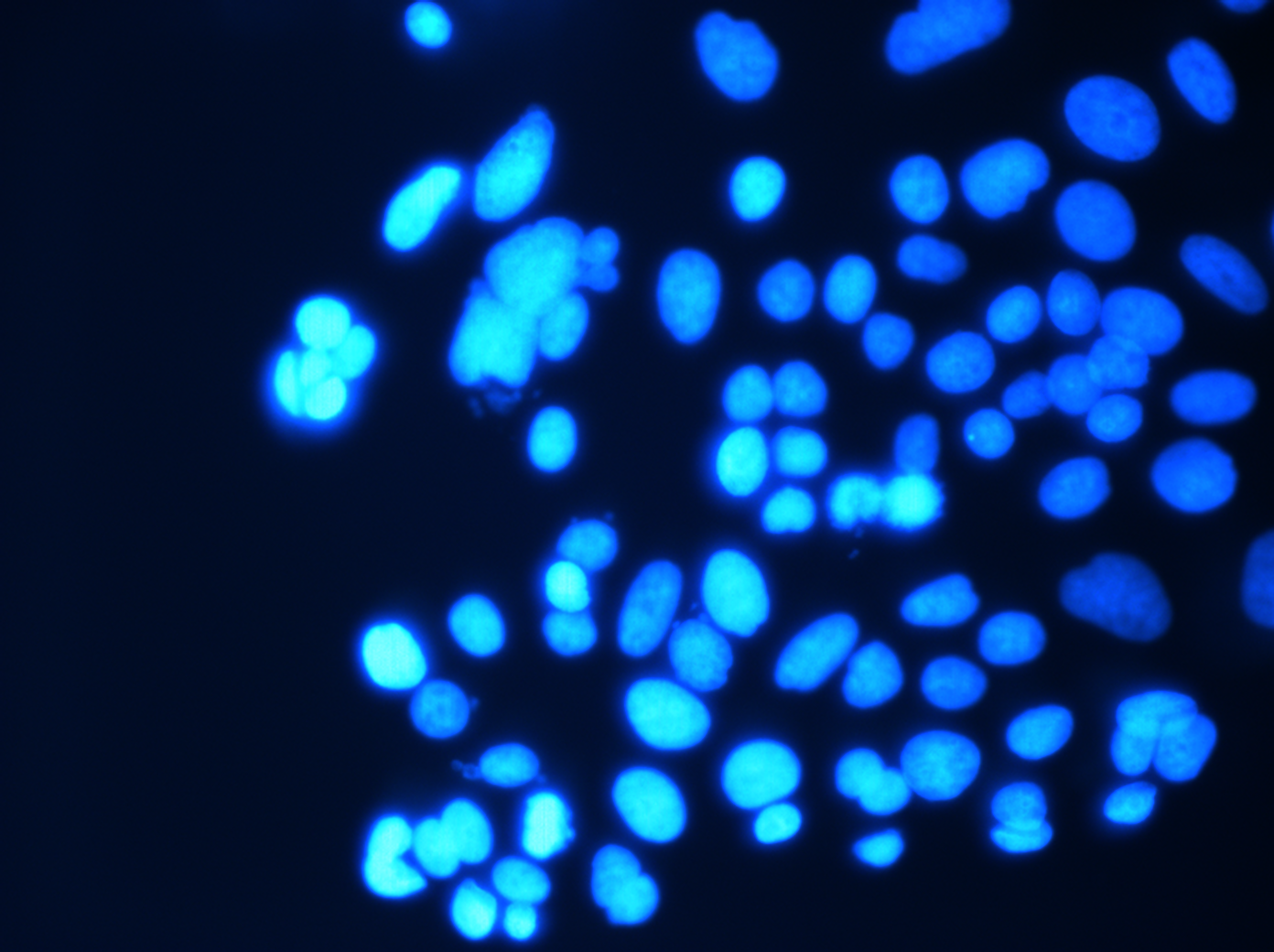

Supplement: S2 Fig — CT-400x-D1. (TIF) [file pone.0155774.s002.tif]

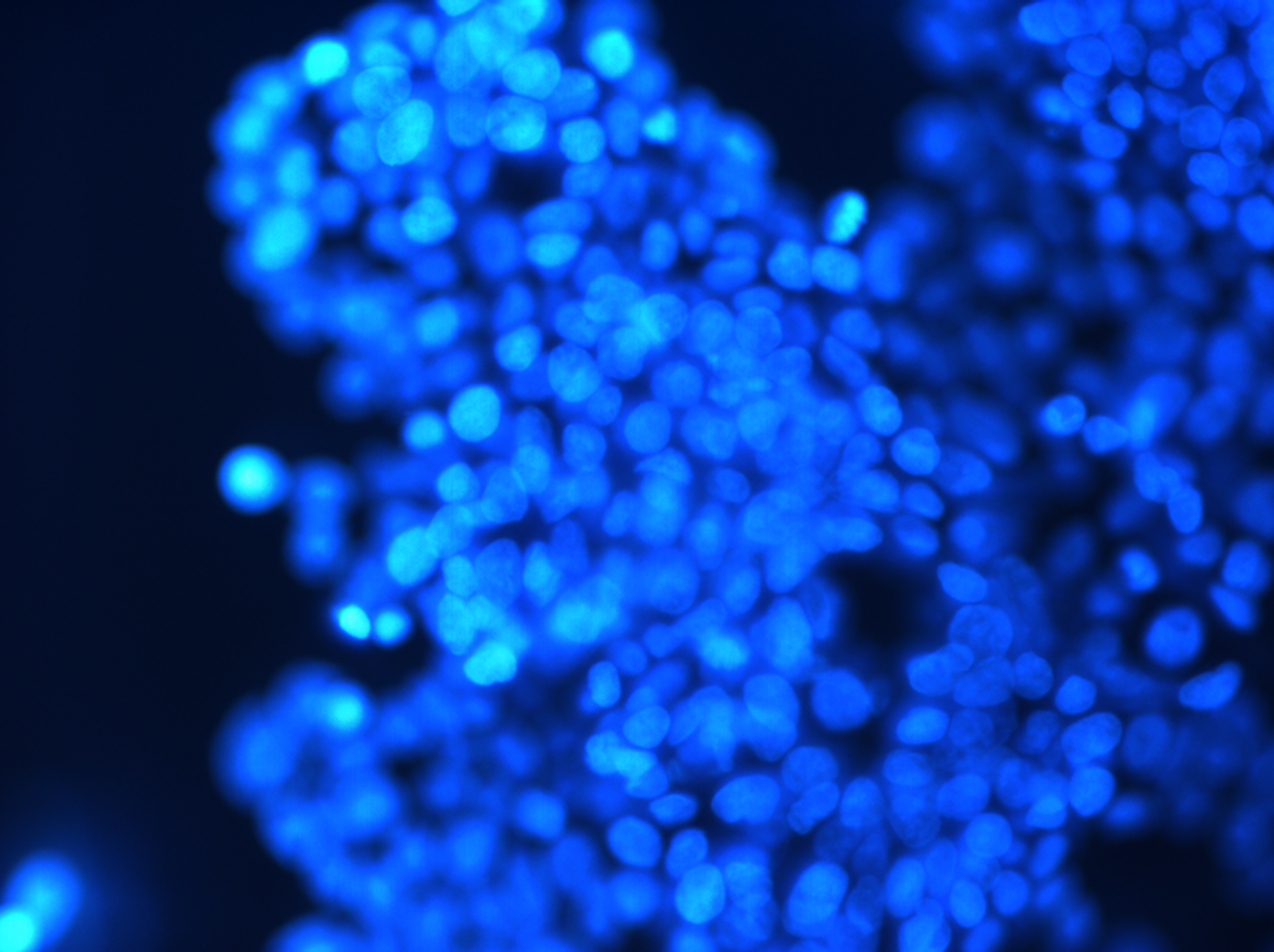

Supplement: S3 Fig — IL-6-CV-400x-D. (TIF) [file pone.0155774.s003.tif]

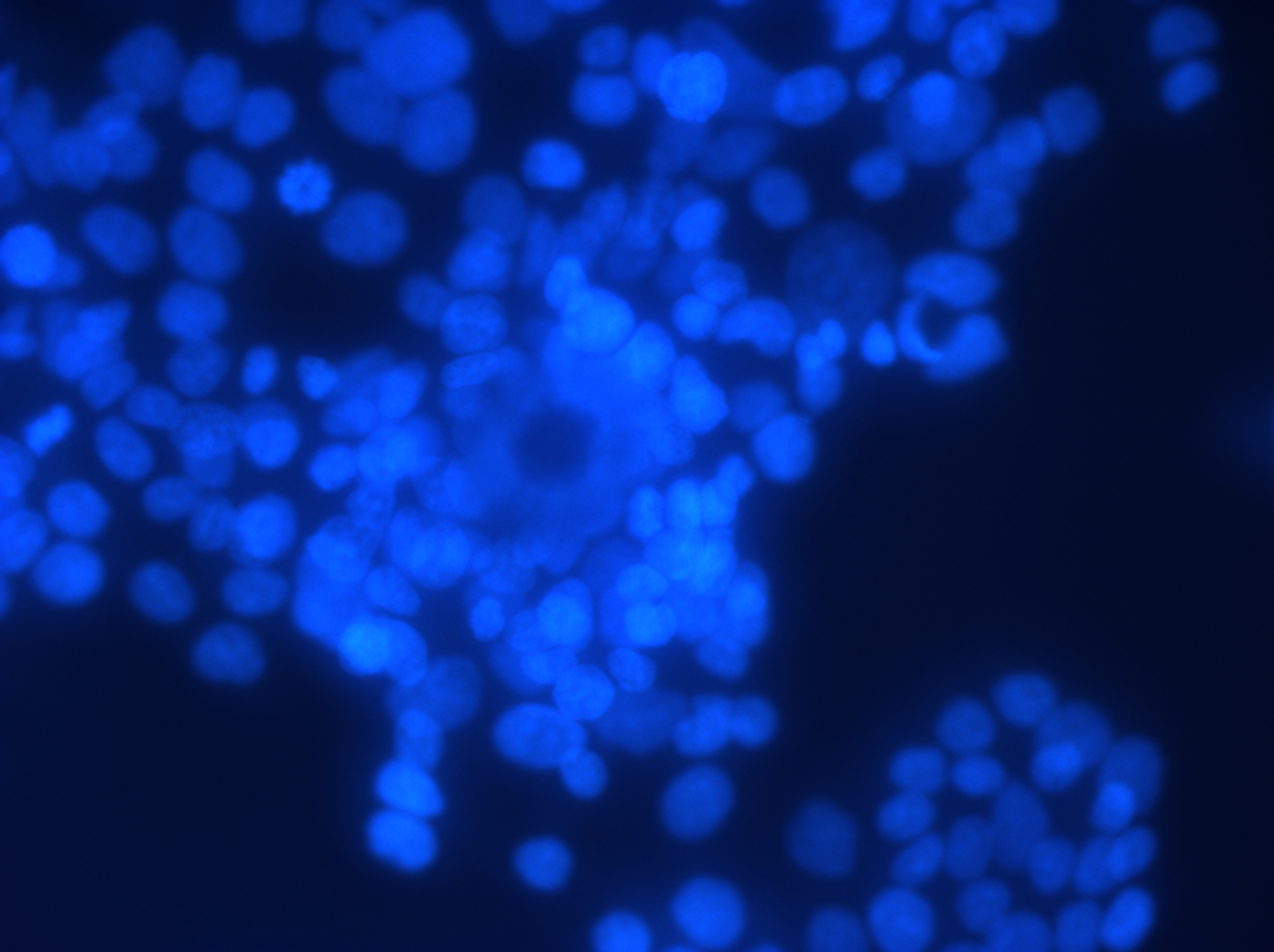

Supplement: S4 Fig — IL-6-SV-400x-D. (TIF) [file pone.0155774.s004.tif]

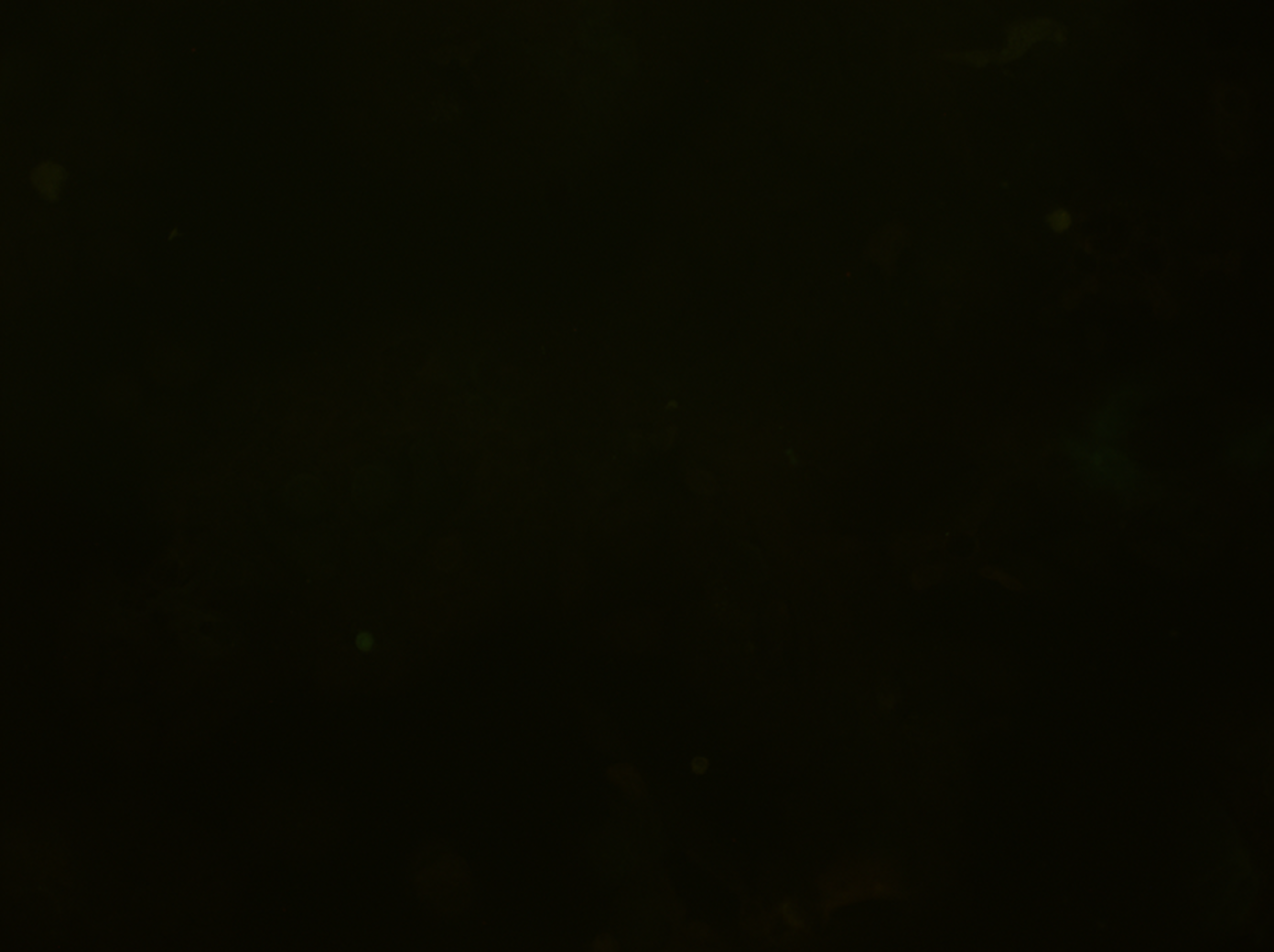

Supplement: S5 Fig — CY-400x-F1. (TIF) [file pone.0155774.s005.tif]

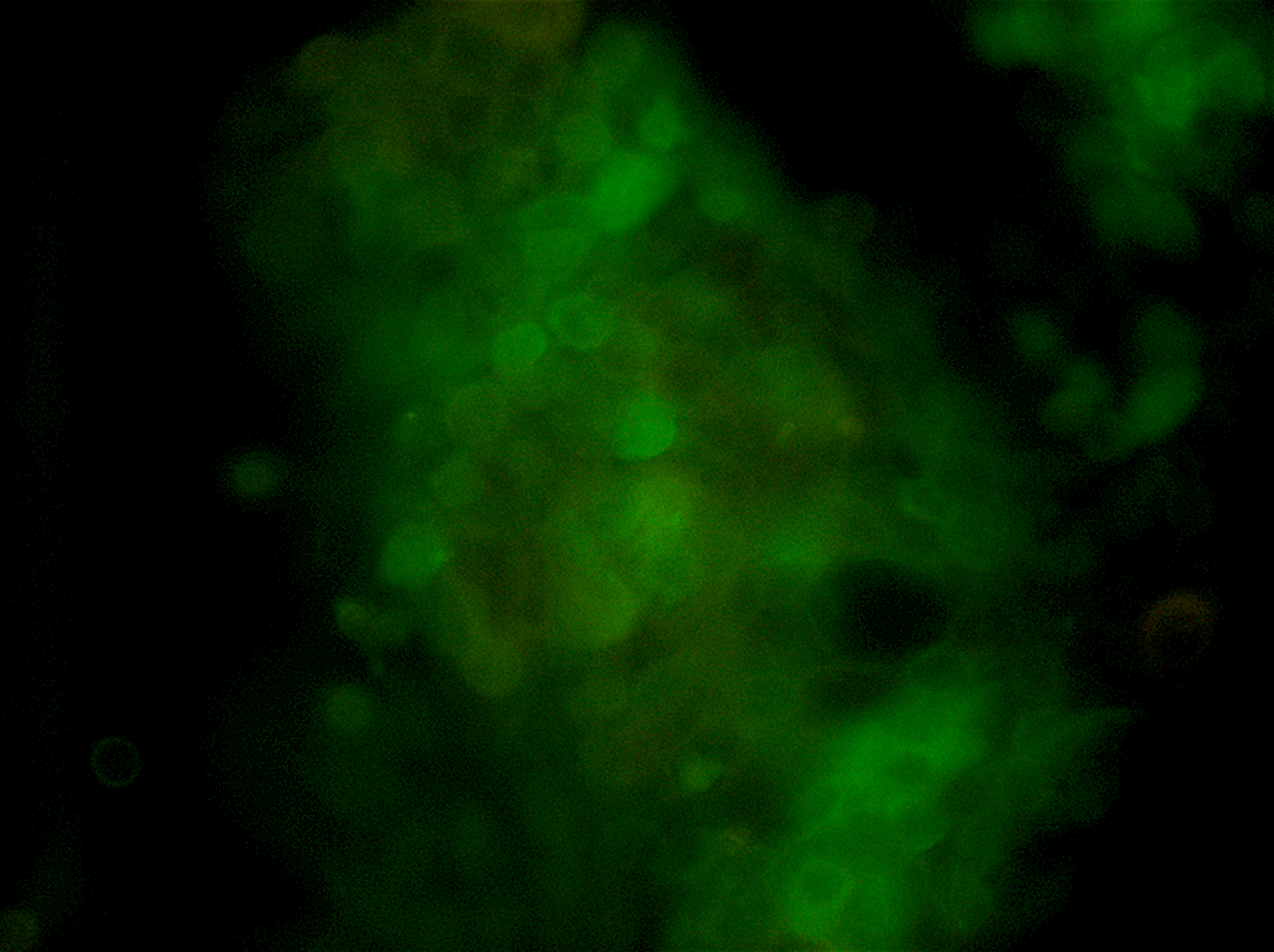

Supplement: S6 Fig — IL-6-VC-400x-F. (TIF) [file pone.0155774.s006.tif]

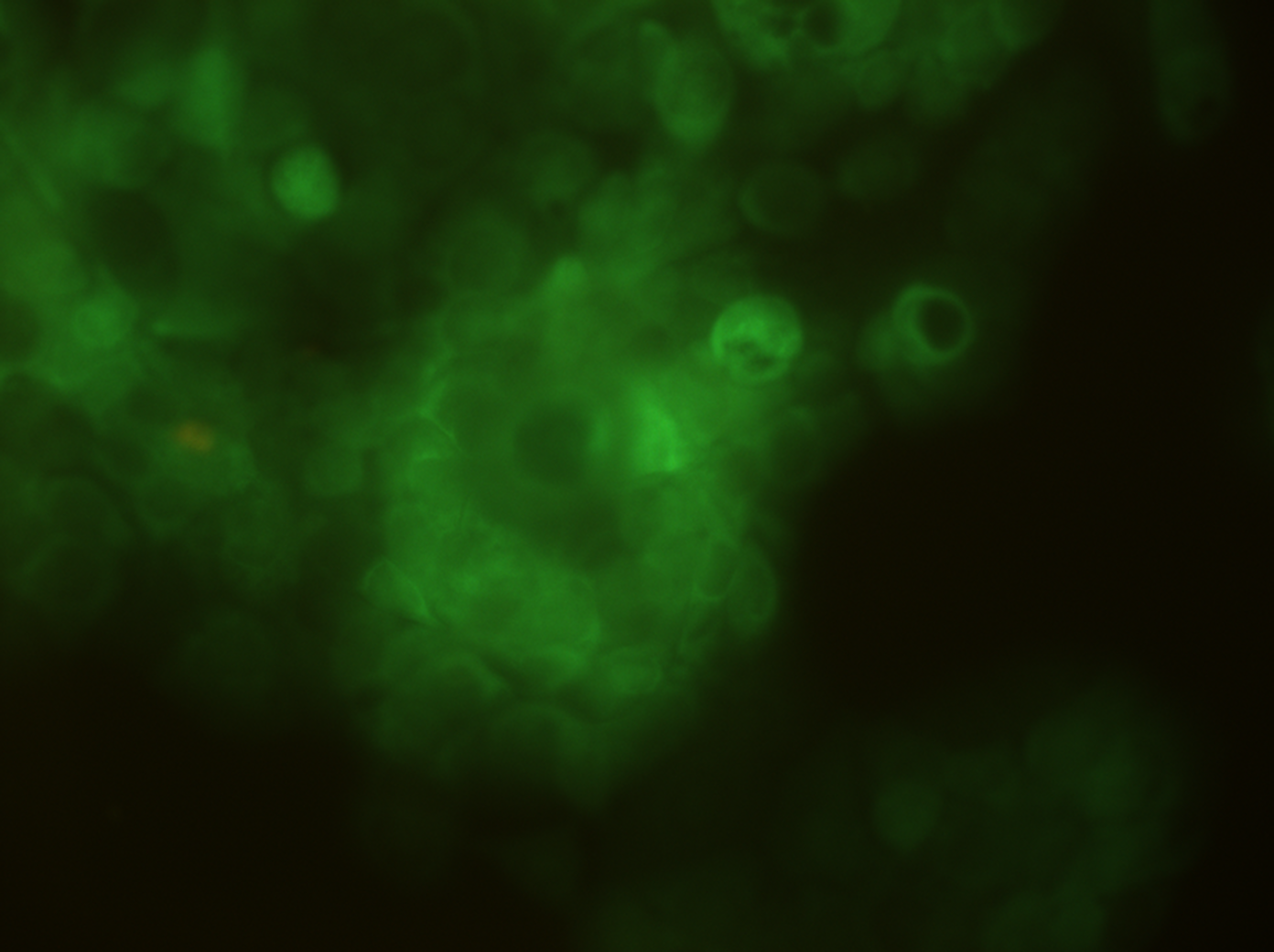

Supplement: S7 Fig — IL-6-SV-400x-F. (TIF) [file pone.0155774.s007.tif]

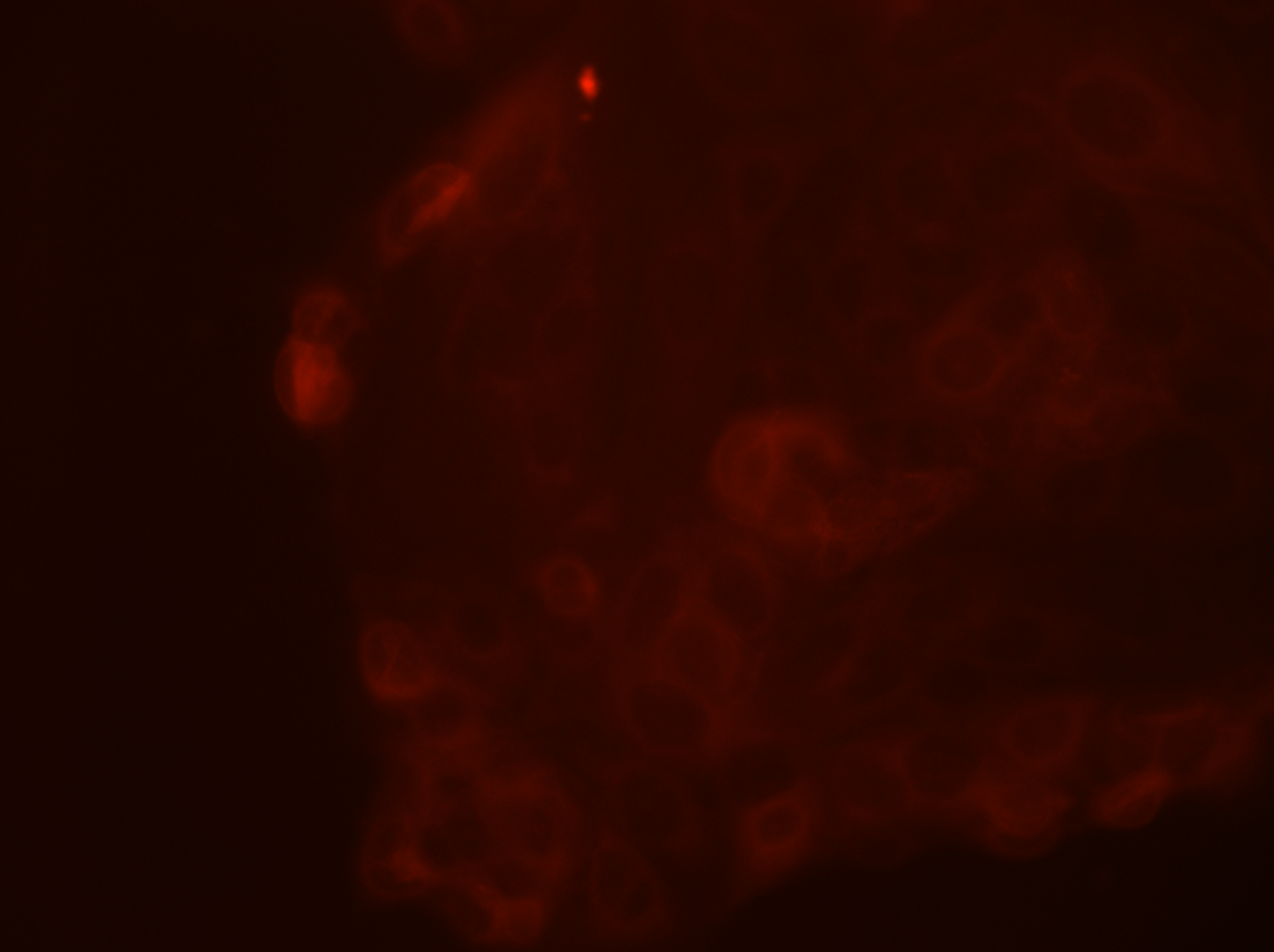

Supplement: S8 Fig — CT-400x-T1. (TIF) [file pone.0155774.s008.tif]

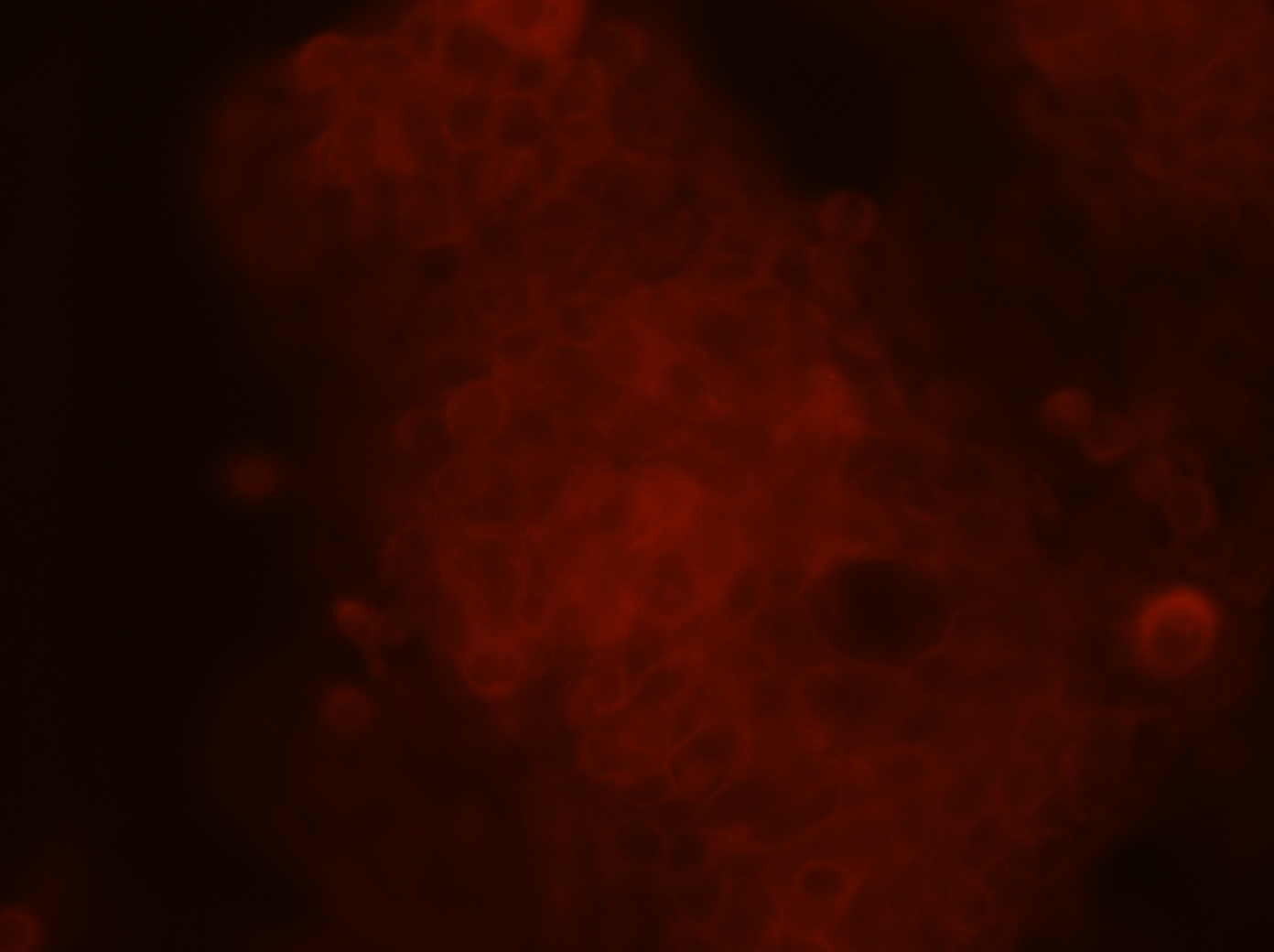

Supplement: S9 Fig — IL-6-CV-400x-T. (TIF) [file pone.0155774.s009.tif]

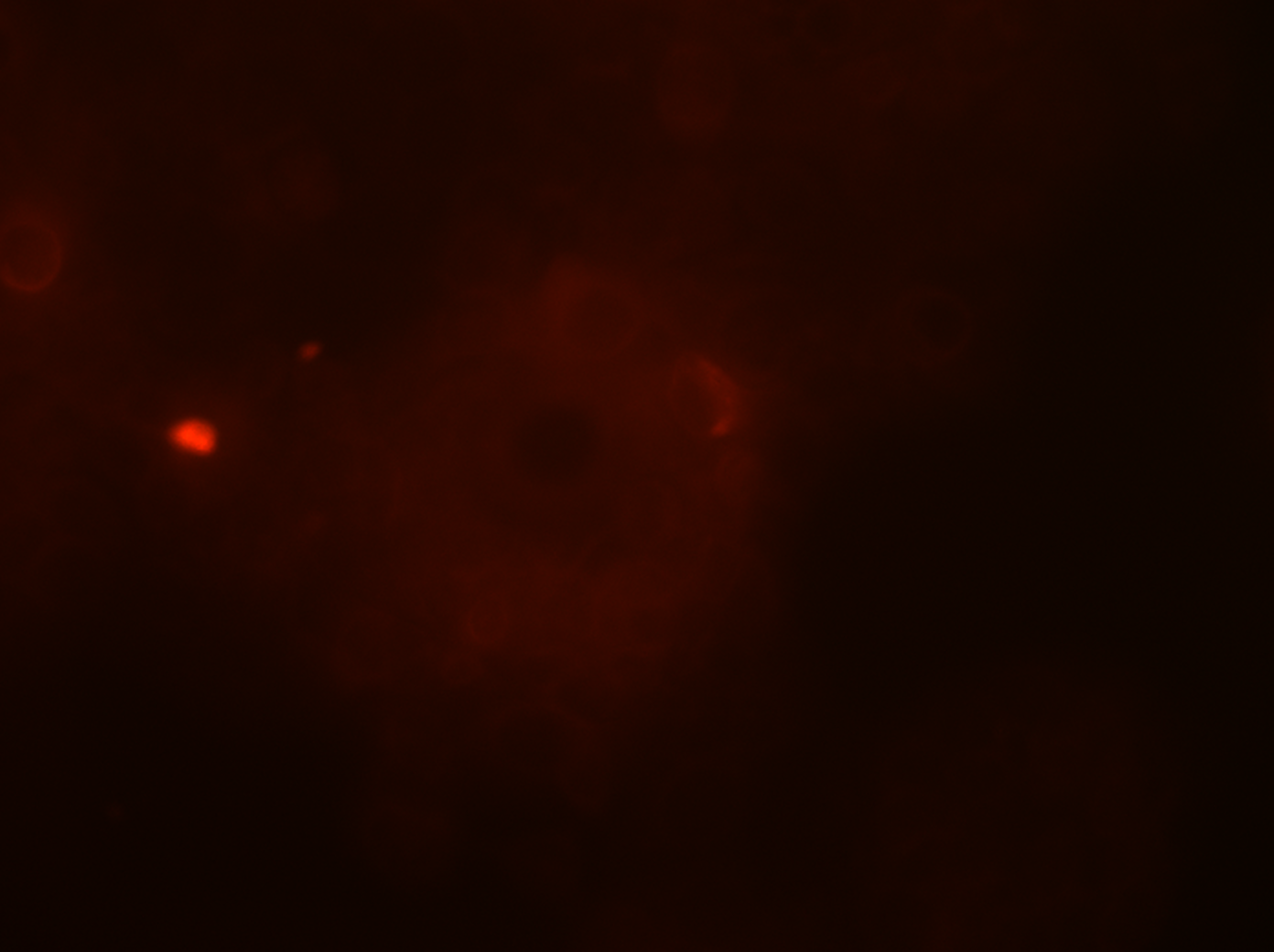

Supplement: S10 Fig — IL-6-SV-400x-T. (TIF) [file pone.0155774.s010.tif]
